# The impact of obesity on ventilator-associated pneumonia, a US nationwide study

**DOI:** 10.1186/s12890-024-02924-y

**Published:** 2024-03-02

**Authors:** Mohamad El Labban, Michella Abi Zeid Daou, Hiba Smaily, Abbas Hammoud, Ghandi Hassan, Syed Khan, Imad Bou Akl

**Affiliations:** 1 Assistant Professor Mayo Clinic College of Science and Medicine-Internal Medicine, Mayo Clinic Health System, Mankato, MN USA; 2 Division of Pulmonary and Critical Care Medicine, Department of Internal Medicine, American University of Beirut, Beirut, Lebanon; 3 Division of Internal Medicine, Department of Internal Medicine, American University of Beirut, Beirut, Lebanon; 4 Faculty of Medicine, American University of Beirut, Beirut, Lebanon; 5 Division of Pulmonary and Critical Care Medicine, Department of Medicine, Mayo Clinic College of Science and Medicine, Mayo Clinic Health System, Mankato, MN USA; 6 Associate Professor of Clinical Specialty-Department of Internal Medicine, American University of Beirut, Beirut, Lebanon

**Keywords:** Obesity, Pneumonia, Ventilator-Associated, Obesity Paradox, Critical care, Respiration, Artificial, Hospital Mortality

## Abstract

**Background:**

Ventilator-associated pneumonia (VAP) is one of the leading causes of mortality in patients with critical care illness. Since obesity is highly prevalent, we wanted to study its impact on the outcomes of patients who develop VAP.

**Methods:**

Using the National Inpatient Sample (NIS) database from 2017 to 2020, we conducted a retrospective study of adult patients with a principal diagnosis of VAP with a secondary diagnosis with or without obesity according to 10th revision of the International Statistical Classification of Diseases (ICD-10) codes. Several demographics, including age, race, and gender, were analyzed. The primary endpoint was mortality, while the secondary endpoints included tracheostomy, length of stay in days, and patient charge in dollars. Multivariate logistic regression model analysis was used to adjust for confounders, with a *p*-value less than 0.05 considered statistically significant.

**Results:**

The study included 3832 patients with VAP, 395 of whom had obesity. The mean age in both groups was around 58 years, and 68% of the group with obesity were females compared to 40% in females in the group without obesity. Statistically significant comorbidities in the obesity group included a Charlson Comorbidity Index score of three and above, diabetes mellitus, hypertension, chronic kidney disease, and sleep apnea. Rates and odds of mortality were not significantly higher in the collective obesity group 39 (10%) vs. 336 (8.5%), *p*-value 0.62, adjusted odds ratio 1.2, *p*-value 0.61). The rates and odds of tracheostomy were higher in the obesity group but not statistically significant. Obese patients were also found to have a longer hospitalization. Upon subanalysis of the data, no evidence of racial disparities was found in the care of VAP for both the obese and control groups.

**Conclusions:**

Obesity was not found to be an independent risk factor for worse outcomes in patients who develop VAP in the intensive care unit.

**Supplementary Information:**

The online version contains supplementary material available at 10.1186/s12890-024-02924-y.

## Background

Obesity is a chronic medical condition characterized by the accumulation of excess body fat. It is associated with an increased risk of adverse health events and mortality [1]. Approximately one-third of adults worldwide were classified as overweight or obese in 2013 [2]. In the United States, the most recent data in 2018 shows that the prevalence of obesity in adults was 42.4%, representing a significant increase from the 30.5% reported in 2000 [3]. This is concerning as there is a multitude of health issues linked to obesity, including but not limited to type 2 diabetes mellitus, cardiovascular disease, obstructive sleep apnea, metabolic dysfunction-associated steatotic liver disease (MASLD) [2, 4].

In the intensive care unit (ICU), the number of obese people getting admitted to ICU for any cause is also increasing and has become a growing concern, with an estimated prevalence of 20% [5]. The association of obesity with worse outcome in critically ill patient needs further studies, and the association depends on the outcome being measured. Obesity is associated with increased ICU stay and morbidity, but its association with mortality is not as clear [6, 7]. The term “obesity paradox” refers to this apparent conflicting correlation between obesity and some patient outcomes. Despite the increased risk in debilitating health conditions, research has shown that obese individuals exhibit better survival rates as compared to their non-obese counterparts in certain acute presentations such as sepsis and acute respiratory distress syndrome (ARDS) [5, 8]. In other words, the paradox is a phenomenon in which obesity appears to be associated with worse outcomes in certain conditions, and with better outcome in others. This paradox has been primarily observed in patients with heart failure [9], end-stage kidney disease [10], ARDS [11], and sepsis [12], but could also apply to a vast number of other conditions. Pneumonia also seems to be one of those conditions that fit the description of the paradox. Obesity was associated with an increased risk of contracting pneumonia but has been shown to be associated with decrease mortality in this population [13, 14].

Ventilator-associated pneumonia (VAP) is a type of pneumonia that develops in a patient more than 48 h after endotracheal intubation [15]. It is estimated that the prevalence of VAP ranges from 5% up to 40% in any given ICU, depending on the setting and diagnostic criteria [16, 17]. It is also known that VAP increases the length of stay, healthcare costs, morbidity, and mortality of ICU patients [15, 18].VAP has several well-known risk factors, such as immunodeficiency, supine position, prolonged mechanical ventilation duration, recent antibiotic use, and gastric colonization by pathogenic bacteria [15, 19]. Very little data exists looking at the relationship between obesity and VAP. Still, studies so far suggest that there is no significant difference in the incidence of VAP between obese and non-obese populations [20, 21]. This indicates that obesity does not seem to be among the many risk factors for VAP. Despite that, obesity’s effect on VAP patients’ outcomes remains far less clear and not nearly as studied. Our paper presents a nationwide study that utilized a large dataset to delve deeper into the impact of obesity on ICU patients with ventilator-associated pneumonia, specifically analyzing whether obesity increases the length of stay, healthcare costs, and mortality rates or whether the obesity paradox also applies to this specific population.

## Materials and methods

### Design and description of the database

We conducted a retrospective cohort study using the national inpatient sample (NIS) from 2017 to 2020. The NIS is part of the Healthcare Cost and Utilization Project (HCUP) and is sponsored by the Agency for Healthcare Research and Quality (AHRQ) [22]. The NIS is the largest inpatient hospital discharge database in the United StatesIt approximates a 20% stratified sample of discharges from US hospitals, excluding rehabilitation and long-term acute care hospitals. The AHRQ has developed linkable files called the Cost-to-Charge Ratio (CCR) that can convert total charges into the actual cost of hospital services. The cost information was obtained from the hospital accounting reports in the Healthcare Cost Report Information System (HCRIS) files collected by the Centers for Medicare & Medicaid Services (CMS). CCR files have hospital-specific cost-to-charge ratios based on an all-payer inpatient or ED costs for most hospitals in the corresponding NIS.

### Data user agreement

Dr El-Labban (first author) completed the data user agreement with HCUP-AHRQ. The HCUP datasets are publicly available and hence are considered exempt from full or expedited institutional review boards (IRB) review (Federal Regulations 45 CFR 46.101 (b).

### Selection of cases and outcome variables examined

In the NIS dataset, the principal diagnosis is the main ICD-10 code of the admission to the hospital and is linked to inpatient status. Final ICD-10 codes are based on final diagnoses after the hospitalization is complete. The secondary diagnosis is a medical condition the patient has on the problem list that could have happened before or during that admission. All procedure codes detected via NIS are linked to the admission. In our study, “ventilator-associated pneumonia (VAP)” was selected as the principal diagnosis (International Classification of Disease, 10th edition, clinical modification [ICD-10-CM]). Our inclusion criteria included adult patients (age 18 years or older) mechanically ventilated for more than 24 h with a principal diagnosis of VAP in the years 2017 to 2020. Although it is usually a complication of an admission, we opted to select VAP as the principal diagnosis. The reason is to accurately capture an admission in which VAP occurred instead of having it as a secondary diagnosis occurring at an unknown timeline. One should keep in mind that the diagnosis of VAP is notoriously variable between hospitals [23]. Patients who were mechanically ventilated for less than 24 h were excluded. There is no specific ICD code for mechanical ventilation < 48 h. ICD-10 codes were also used to identify secondary diagnoses mentioned in supplemental Table 1. Patients’ co-morbidities were also described through the Charlson Comorbidity Index. Outcomes, including mortality, tracheostomy, length of stay, and total charges, were generated from the NIS dataset. We classified obesity as the following: mild (BMI 30–40), moderate (BMI > 40), and severe (BMI > 50).

### Statistical analysis

Statistical analyses were conducted using STATA BE Version 17.0. All statistical tests were two-sided, and a *p*-value of < 0.05 was considered to be statistically significant. Chi-square analysis was used to describe the difference in patients’ characteristics and secondary diagnoses according to the presence and absence of obesity. The impact of obesity on outcomes was described using Chi-square analysis to compare outcomes’ rates and multivariable regression models to describe the isolated impact of obesity on the odds of the outcome. The following variables were included in the regression model: age, female gender, race (White (as the reference), Black, Hispanic, Asian/Pacific, Native American, other), Charlson Comorbidity Index as categories (Group 0 (Score of zero, this was the reference), Group 1 (Score of 1), group 2 (Score of 2), group 3 (score of ≥ 3), Insurance status (Medicare (reference), Medicaid, Private insurance, self-pay), Diabetes Mellitus II, Hypertension, Chronic kidney disease, Supraventricular tachycardia, sepsis, sleep apnea (including hypovention syndromes), and chronic obstructive pulmonary disease (COPD). In the subanalysis, Pearson’s χ 2 test analysis was used to describe the difference in primary and secondary outcomes across the six race categories. All the regression models in our study had a significant F-value (Prob > F < 0.01), which in turn confirms that the independent variables reliably predict the studied variable.

## Results

### Demographic characteristics

The studied population included 4,227 patients with ventilator-associated pneumonia, 3832 (91%) of whom were not obese and 395 (9%) were. The patients with obesity were divided into three categories according to their body mass index (BMI): 155 had a BMI of 30–40 kg/m2(3.5%), 145 had a BMI of 40–50 kg/m2(3.3%), and 95 had a BMI > 50 kg/m2(2.2%). Patients in both groups were around 58 years old. Obese patients were more likely to be females (65% vs. 40%), white (55% vs. 47%), and covered by Medicare (69% vs. 59%). The percentage of people with a score of 3 or greater was higher in the obese group than in the non-obese group (56% vs. 42%, respectively; *p*-value < 0.01). Obese patients were more likely to have hypertension (76% vs. 56%, *p*-value < 0.01), diabetes mellitus (62% vs. 36%, *p*-value < 0.01) chronic kidney disease (27% vs. 14%, *p*-value < 0.01), and sleep apnea (18% vs. 3%, *p*-value < 0.01) (Table 1).

**Table 1:** Patient characteristics

Characteristics	Without obesity	With obesity	*p*-value
no (%)	3832 (91)	395 (9)	
BMI categories, no (%)			
30–40		155 (3.5)	
40–50		145 (3.3)	
Above 50		95 (2.2)	
Year			0.7
2017	1022 (26.6)	85 (21.5)	
2018	1008 (26.3)	100 (25.3)	
2019	1066 (27.8)	120 (30.3)	
2020	736 (19.1)	90 (22.7)	
Female, no (%)	1533 (40)	268 (68)	< 0.01
Age (y)	58.5	58.3	
Race, no (%)			0.35
White	1801 (47)	217 (55)	
Black	1073 (28)	126 (32)	
Hispanic	460 (12)	31 (8)	
Asian or Pacific Islander	176 (4.6)	11 (3)	
Native American	34 (0.9)	0	
Other	249 (6.5)	11 (3)	
Charlson Comorbidity Index score, no. (%)			< 0.01
0	536 (14)	15 (4)	
1	766 (20)	43 (11)	
2	920 (24)	114 (29)	
>=3	1609 (42)	221 (56)	
Insurance type, no. (%)			0.2
Medicare	2261 (59)	272 (69)	
Medicaid	1111 (29)	71 (18)	
Private Insurance	421 (11)	51 (13)	
Self-pay	23 (0.6)	0	
Comorbidities, no. (%)			
Sepsis	1686 (44)	165 (42)	0.6
DMII	1380 (36)	244 (62)	< 0.01
HTN	2146 (56)	300 (76)	< 0.01
CKD	536 (14)	106 (27)	< 0.01
SVT	728 (19)	106 (27)	0.1
Sleep apnea*	115 (3)	71 (18)	< 0.01
COPD	651 (17)	59 (15)	0.71

BMI: Body Mass Index, DM II: Diabetes Mellitus II, HTN: Hypertension; CKD: Chronic Kidney Disease; SVT: supraventricular tachycardia; COPD: Chronic obstructive pulmonary disease

*Sleep apnea: Including obstructive sleep apnea, central sleep apnea, hypoventilation syndromes

### Primary outcome

There was no statistically significant difference in the mortality rates among obese and non-obese patients (10% vs. 8.5%, *p* value = 0.62) (Table 2). The mortality rate was higher in the BMI groups 40–50 (10%) and above 50 (16%) but lower in the BMI 30–40 group (6.4%). The regression analysis showed higher odds of mortality in the obesity group. However, that was not statistically significant (adjusted OR 1.2, *p*-value 0.61). Comparable results were seen in the BMI groups 30–40 and 40–50 (adjusted OR 0.73, *p*-value 0.71, adjusted OR 0.99, *p*-value 0.9). Lower odds of mortality were noted in the BMI above 50 group yet still statistically insignificant (adjusted OR 2.96, *p*-value 0.18).

**Table 2:** In-hospital mortality rates and odds

	Total patients who died	Without obesity	With obesity	*p*-value	Adjusted Odds Ratio	Confidence Interval	*p*-value
Obesity, no (%)	375	336 (8.5)	39 (10)	0.62	1.48	0.64–3.43	0.35
BMI 30–40, no (%)	343		10 (6.4)		0.73	0.13–3.84	0.71
BMI 40–50, no (%)	343		14 (10)		0.9	0.32–3.01	0.99
BMI above 50, no (%)	341		15 (16)		2.96	0.59–14.7	0.18

### Secondary outcomes

There was no statistically significant difference in the tracheostomy rates among obese and non-obese patients (1.27% vs. 0.25%, *p* value = 0.14) (Table 3). Patients with a 30–40 kg/m2 BMI group had a 3.2% tracheostomy rate. No tracheostomy procedures were noted in patients with a BMI of 40 and above. The adjusted odds ratio of having a tracheostomy was higher in the obese group but statistically insignificant (adjusted OR 5.95, *p*-value 0.16).

**Table 3:** In-hospital tracheostomy rates and odds

	Without obesity	With obesity	*p*-value	Adjusted Odds Ratio	Confidence Interval	*p*-value
Obesity, no (%)	10 (0.25)	5 (1.27)	0.14	6.89	0.59–80.3	0.12
BMI 30–40, no (%)	-	5 (3.2)	-	12.8	0.34–480	0.16

Obese patients had a longer length of stay in the hospital (12 vs. 11.2 days) (Table 4). The linear regression analysis showed mean adjusted difference of 1.4 more days; however, it was not statistically significant (*p*-value 0.38). The length of stay of patients with BMI 30–40 kg/m2 was significantly shorter than non-obese patients (8 days vs. 11.2 days, adjusted means − 2.9 days, *p*-value < 0.01). Patients with a BMI of 40–50 kg/m2 had a more extended stay in the hospital than those with a normal BMI (17.9 vs. 11.2 days, adjusted means = + 7.4 days, *p*-value = 0.03).

Obese patients had a higher total charge for their admission ($166,876 vs. $150,930, adjusted means + 27,620 $, *p*-value = 0.3) (Table 4). Patients with a BMI of 40–50 kg/m2 ($260,697 vs. $150,930, adjusted means = + 120,190, *p*-value = 0.05). On the other hand, charges were lower in patients with BMI > 50 kg/m2 ($122,920 vs. $150,930, adjusted means = -23,748$, *p* = 0.41) and BMI 30–40 kg/m2 ($106,048 vs. $150,930, adjusted means = -35,951$, *p*-value = 0.08).

**Table 4:** Length of stay and total admission charges

	Without obesity	With obesity	Adjusted Means	*p*-value
Obesity, days	11.2	12	1.31	0.4
BMI 30–40, days	-	8	-2.9	< 0.01
BMI 40–50, days	-	17.9	7.45	0.03
BMI above 50, days	-	9.6	-1.38	0.53
Obesity, $	150,930	166,876	30,564	0.26
BMI 30–40, $	-	106,048	-35,951	0.08
BMI 40–50, $	-	260,697	120,190	0.05
BMI above 50, $	-	122,920	-23,748	0.41

### Rates of in-hospital mortality, tracheostomy, length of stay, and total charge by race and ethnicity

The primary and secondary outcomes were further analyzed by race and ethnicity. Obese patients with VAP were noted in all races except Native American. There were no differences of statistical significane among the six races in the obese and non-obese group. In-hospital mortality in the obese group was highest in Black (16%) and Hispanic (16%) patients and in Native Americans (28.5%) in the non-obese group (Table 5). In-patient tracheostomy was only noted in five obese patients all of whome were black. Race was not found to be an independent risk factor for worse mortality outcomes in obese and non-obese patients with VAP (Table 6).

**Table 5:** Inpatient Mortality, Tracheostomy, Length of Stay, and Total Charge by Race and Ethnicity

Outcomes	White		Black		Hispanic		Asian/Pacific		Native American		Other		*p* value	
	Obese group	Control group	Obese group	Control group	Obese group	Control group	Obese group	Control group	Obese group	Control group	Obese group	Control group	Obese group	Control group
Mortality, no. (%)	15 (7)	159 (8.7)	20 (16)	81 (7.4)	4 (16)	41 (8.51)	0 (0)	15 (8.57)	0 (0)	10 (28.5)	0 (0)	30 (12)	0.7	0.41
Tracheostomy, no. (%)	0 (0)	5 (0.27)	5 (4.17)	0 (0)	0 (0)	0 (0)	0 (0)	0 (0)	0 (0)	0 (0)	0 (0)	5 (2)	0.7	0.29
Length of stay (days)	11.9	10.8	12.79	11.6	9.6	12	13.5	10.57	-	8.2				
Total Charge ($)	161,861	144,738	169,480	136,823	123,915	179,374	517,097	163,010	-	125,665				

**Table 6:** Adjusted odds ratio of Mortality by Race and Ethnicity

	Obese group	*P*-value	Confidence Interval	Control group	*P*-value	Confidence Interval
White	Reference			Reference		
Black	4.91	0.19	0.43–55.3	0.73	0.35	0.38–1.41
Hispanic	1.36	0.8	0.1-17.08	1.21	0.64	0.52–2.83
Asian/Pacific	-	-	-	0.9	0.9	0.27–3.14
Native American	-	-	-	1.19	0.68	0.5–2.81

## Discussion

The relationship between obesity and the outcomes of patients with VAP remains unclear. Research on the subject is still scarce, with conflicting results so far. Our study found no significant differences in outcomes between obese and non-obese ICU patients with ventilator-associated pneumonia.

In our study, half of the 4,227 patients with ventilator-associated pneumonia, half of the included patients were of white ethnicity. As per the National Institutes of Health (NIH), obesity affects 17.4 to 42% of the general population, depending on the race. However, in our study, only up to 11% of the patients per ethnic group with VAP were classified as obese. Obesity was noted in 9% of our overall population, with a higher proportion of obesity among women. In contrast, when examining the NIH statistics for the general population, 42% of adults were obese in the USA, with a predominance of obesity among men. Additionally, 56% of obese compared to 42% of non-obese patients, had a Charlson Comorbidity Index score of 3 and more, suggesting a higher burden of comorbidities compared to the non-obese group. Specifically, diabetes, hypertension, and chronic kidney disease were significantly more prevalent among obese patients. A recent retrospective analysis using the NIS captured 33,140 VAP diagnoses [24]. The authors likely used VAP as a secondary rather than a primary diagnosis. Although they mentioned the principal diagnostic categories of hospitalizations with VAP, since the latter is a secondary diagnosis, it would be challenging to determine whether it occurred during the referenced principal diagnosis or in a previous admission. In their study, obesity was found in 18.1% of cases, but they did not categorize obesity according to different BMI classes. They also did not report the impact of obesity on mortality. Even with a smaller sample size of VAP hospitalizations, our study stands out for several reasons. We classified obesity based on BMI and reported its impact as an independent factor on mortality (adjusted odds ratio). Additionally, we investigated the impact of obesity on in-patient tracheostomy, an indicator of ventilator liberation, which has not been described in VAP hospitalizations before.

We found that obesity did not have a significant effect on mortality in ICU patients with VAP. Though the results were not statistically significant (10% vs. 8.5%, *p*-value 0.61, adjusted OR 1.2, *p*-value 0.61), obese patients had higher mortality rates. The fact that the adjusted odds ratio was not significant implies the observation of higher mortality in obese patients is not due to obesity itself but rather to its associated comorbidities. This throws doubt on the understanding that obesity is an advantage in the ICU. Obesity has traditionally been associated with lower mortality in ICU patients [7, 25–27]. Although the potential protective mechanisms are not yet fully understood, it has been suggested that the increased energy reserves stored in the excess adipose tissue in obesity may confer an advantage in short-term high-stress situations. Additionally, adipose tissue is now recognized to play more complex roles beyond simple energy storage; these include the release of hormones like leptin and adiponectin, as well as inflammatory mediators like TNF-alpha, IL-6, resistin, and visfatin, which all play essential roles in metabolism and inflammation [7, 28]. On the other hand, some studies have found that obesity is associated with higher mortality in the ICU population, as one might expect [29, 30].

Obesity is associated with prolonged duration of mechanical ventilation. The standard practice suggests performing a tracheostomy after prolonged intubation, and institution haves different policies guiding tracheostomy placement as clear-cut recommendations are lacking. Consequently, one might expect a higher likelihood of tracheostomy among obese compared to non-obese patients. Our study echoed this theory, as we found that rates of tracheostomy were higher in the obese group (5 (1.27%) vs. 10 (0.25%), *p*-value 0.14). This result was not statistically significant, and as such, obesity was not found to be a risk factor for long-term respiratory failure and need for tracheostomy. Our study is the first to address the rate of tracheostomy in obese patients with VAP.

A recent meta-analysis by Peres et al. exploring the risk factor for longer length of stay (LOS) found that mechanical ventilation, hypomagnesemia, delirium, and malnutrition were the most significant variables to have an effect [31]. Although examined in 7 of the 89 papers studied, BMI did not show a consistent effect [31]. In our study, the average LOS stay was 11.2 days in non-obese patients vs. 12 days in the obese group. Those with mild and extreme obesity had a shorter stay (8 & 9.6 days, respectively). Paradoxically, patients with a BMI of 40–50 had a longer LOS (17.9 days). In the linear regression analysis, we found that mild obesity contributed to a shorter LOS by 2.98 days on average, as opposed to moderate obesity, which elongated the LOS by an adjusted mean of 7.45 days (*p*-value < 0.05). Such inconsistency in the results among the obesity subgroups calls for future studies on the topic of LOS.

We found that the obese group had a higher total admission charge (150,930 vs. 166,876 $). Similar to the LOS, the subgroup analysis had conflicting results. Patients in the BMI group 30–40 and above 50 had a lower total charge (122,920 & 106,048, respectively). The result was higher in the moderately obese group (260,697). In the linear regression analysis none of the BMI subgroups significantly impacted the total charge as an independent factor (Table 4). Direct studies that seek to analyze the effect of obesity on the cost of hospitalization of patients with ventilator-associated pneumonia are non-existent; our study is the first to our knowledge. It is difficult to draw a direct association between obesity, cost, and ventilator-associated pneumonia as our research does not account for other reasons for hospitalization and their potential impact on the cost. However, it remains important to note that mild obesity was shown to have the least overall cost of hospitalization, likely due to fewer comorbidities complicating the hospital stay, in concordance with the estimated BMI-related medical expenditure reported by Ward et al. [32].

We found that obesity did not have a clear protective or harmful impact on patients with VAP. Such results neither support nor refute the obesity paradox theory. Several theories can explain the reasoning behind such findings. First, many studies in the past have adjusted for ICU admission categories like medical, surgery, and trauma [7, 25, 26]. However, these broad categorizations and varieties may exist within each one. Even if we were to hypothesize that obesity has some effect on the outcomes of ICU patients, it cannot be assumed that it would have the same impact on all types of ICU patients (those with sepsis, ARDS, heart failure, VAP, etc.). Second, complex confounders such as quality of care can provide the obese group with advantages and disadvantages at the same time. Quality of care depends on factors like geographic location, socioeconomic status, insurance type, hospital quality, aggressiveness of treatment, and systemic biases against obese patients. For example, Arabi et al. found that the decreased mortality in obese sepsis patients can be explained by more aggressive sepsis interventions in the obese [33]. On the other hand, it has been known for some time that obesity may negatively impact the attitudes of healthcare professionals toward patients and the quality of care they receive [34–36]. Unfortunately, such confounders are very difficult to account for. Our study looked at insurance type, which was not significantly different between the obese and non-obese groups.

Numerous prior studies demonstrate the presence of racial disparities in the healthcare system in the United States. For example, black patients are noted to have higher rates of emergency department visits for both asthma [37] and heart failure [38]exacerbations. According to a systematic review published in Critical Care Medicine, Black patients were found to have higher mortality rates [39]. However, this effect was attributed to age, severity of illness, hospital type, and socioeconomic status rather than race as an independent factor. Our data shows that Black and Hispanic obese patients with VAP had higher mortality rates, but their respective adjusted odds ratios were not statistically significant. Therefore, similar to the data from McGowan et al., the difference in mortality rates is likely related to confounders rather than race. In the non-obese group, Native Americans with VAP had the highest rate of mortality compared to the other races; however, the adjusted odds ratio was again statistically insignificant.

There are some limitations to our study. We conducted a retrospective cohort study using the NIS, an administrative database that limits the uniformity of VAP diagnosis with potential misclassification secondary to using the ICD-10 CM codes. The diagnosis of VAP is extremely variable, enough to render comparisons between hospitals very limited, even when standardized cases eliminate variability in clinical data abstraction [23]. Second, because of the administrative nature of our database, our analysis did not include the severity of disease at ICU admission or the types, dosages, and frequencies of different VAP-specific treatments during ICU stay. Third, we could not consider different measures of adiposity like waist circumference and waist-to-hip ratio, height estimates, or weight fluctuations during ICU stay. The primary cause of mortality is not specified in NIS; therefore, we presented the total all-cause mortality. Our study continued into 2020, during the time when the COVID-19 pandemic had already begun. Unfortunately, COVID-19 cases were underreported, which limits our ability to accurately determine the frequency of infections. To address this limitation, we conducted a subanalysis over a four-year period. The results indicate no significant difference in the number of VAP patients between the two groups across the four years (*p*-value 0.7, Table 1).Since this is not a prospective study, the conclusion that obesity does not significantly impact the outcomes of VAP should be viewed with caution. Despite these limitations, this study is an important contribution to the current understanding of the obesity paradox. We investigated how obesity impacted rates and odds of tracheostomy and the total hospital charges, both of which are not traditionally included in studies of the obesity paradox. We also are the first to report data on racial disparities in the outcomes of VAP in both the obese and non-obese cohorts.

## Conclusion

Patients with and without obesity – who have ventilator-associated pneumonia – have similar mortality rates, tracheostomy rates, length of stay at the hospital, and hospital costs and charges. Traditionally, obesity has been thought to be associated with worse outcomes in critically ill patients, while evidence from recent studies suggests a paradox. Our results neither support the traditional nor the paradoxical impact of obesity on patients with VAP.

### Electronic supplementary material

Below is the link to the electronic supplementary material.

Supplementary Material 1

## Data Availability

The datasets used and/or analyzed during the current study available from the corresponding author on reasonable request. The dataset used contains deidentified data.

## Declarations

### Diversity

The authors of this study are diverse in their gender, nationality, practice locality, practice type, religion, and race.

## Abbreviations

NAFLD: non-alcoholic fatty liver disease
ICU: Intensive Care Unit
ARDS: Acute Respiratory Distress Syndrome
VAP: Ventilator-associate pneumonia
NIS: National inpatient sample
HCUP: Healthcare Cost and Utilization Project
AHRQ: Agency for Healthcare Research and Quality
IRB: Institutional review boards
ICD: International Classification of Disease
BMI: Body mass index
OR: Odds ratio
NIH: National Institutes of Health
TNF: Tumor necrosis factor
IL: Interleukin
