# Supplementary material for: The impact of obesity on ventilator-associated pneumonia, a US nationwide study
Source: BMC Pulm Med. 2024 Mar 2;24:104. doi: 10.1186/s12890-024-02924-y (PMC10908123; doi:10.1186/s12890-024-02924-y)
Supplement: Supplementary file 1 — Supplementary Material 1 [file 12890_2024_2924_MOESM1_ESM.docx]

Supplemental Table 1 Diagnosis and corresponding ICD-10 codes

| Diagnosis | ICD-10 |
| --- | --- |
| VAP | J95851 |
| Sepsis | H594,T802,T826,T827,T835,T836,T845,T847,T857,O860,R5082,K6811,T880,T802,R7881,A400,A401,A403,A408,A409,A427,A427,B377,A267,A282,A5486,B007,A327,A241,A392,A394,A207,A217,A483,R578,A419,A4101,A4102,A411,A412,A414,A4150,A4151,A4152,A4153,A4159,A4181,A4189,A419 |
| Diabetes Mellitus type II | E1100,E1101,E1111,E1122 ,E1129 ,E1131,E11311,E11319,E11321,E113211,E113212,E113213, E113219,E113291,E113292,E113293,E113299,E113311,E113312,E113313,E113319,E113391,E113392,E113393,E113399,E113411,E113412, E113413,E113419,E113491,E113492,E113493,E113499,E113511,E113512,E113513,E113519,E113521,E113522,E113523,E113529,E113531,E113532 ,E113533,E113539,E113541,E113542,E113543 , E113549,E113551,E113552,E113553,E113559 ,E113591,E113592,E113593,E113599,E1136,E1137X1,E1137X2,E1137X3,E1137X9 ,E1139,E1140 ,E1141,E1142,E1143, E1144,E1149,E1151,E1152,E1159,E1161,E11610 ,E11618,E11620,E11621, E1162,E11628,E11630 ,E11638,E11641,E11649 ,E1165,E1169, E118,E119 |
| Essential Hypertension | I110,I119,120 ,I129 ,I1310,I1311,I132 ,I130 ,I150 ,I151, I152 , I158 ,I159 ,I160 ,I161 , I169 |
| Supraventricular tachycardia | I471,479,I480,I4811,I4819,I4820, I4821, I483, I484, I4891, I4892 |
| Chronic kidney disease | N181, N182, N1830, N1831, N1832, N184, N185, N18, N189 |
| Tracheostomy | 0B110Z4, 0B114F4, 0B114Z4 |
| Sleep apnea/hypoventilation syndromes | G4730, G4731, G4733, G4734, G4736, G4737, G4739 |
| Chronic obstructive pulmonary disease | J410, J411, J418, J42, J430, J431, J432, J438, J439, J449, J479 |
